# Capacity gaps in health facilities for case management of intestinal schistosomiasis and soil-transmitted helminthiasis in Burundi

**DOI:** 10.1186/s40249-018-0447-y

**Published:** 2018-07-04

**Authors:** Paul Bizimana, Katja Polman, Jean-Pierre Van Geertruyden, Frédéric Nsabiyumva, Céline Ngenzebuhoro, Elvis Muhimpundu, Giuseppina Ortu

**Affiliations:** 1 Global Health Institute, Department of Epidemiology and Social Medicine, Faculty of Medicine and Health Sciences, University of Antwerp, Gouverneur Kinsbergencentrum, Doornstraat 331, Wilrijk, 2610 Antwerp, Belgium; 2 Département des Sciences de la Santé Publique, Direction de la Formation, Institut National de Santé Publique, B.P, 6807 Bujumbura, Burundi; 3 Département de Médecine Communautaire, Faculté de Médecine de Bujumbura, Université du Burundi, Bujumbura, Burundi; 4 Medical Helminthology Unit, Department of Biomedical Sciences, Institute of Tropical Medicine, Antwerp, Belgium; 5 Département de Médecine Interne, Faculté de Médecine de Bujumbura, Université du Burundi, Bujumbura, Burundi; 6 Département des Sciences de la Santé Publique, Institut Universitaire des Sciences de la Santé et de Développement Communautaire, Bujumbura, Burundi; 7 Programme National Intégré de Lutte contre les Maladies Tropicales Négligées et la Cécité, Département des programmes de santé, Ministère de la Santé Publique et de la Lutte contre le Sida, Bujumbura, Burundi; 8 Department of Infectious Diseases and Epidemiology, Schistosomiasis Control Initiative, Imperial College, London, UK

**Keywords:** Capacity gap, Health facility, Case management, Intestinal schistosomiasis, Soil-transmitted helminthiasis, Burundi

## Abstract

**Background:**

Schistosomiasis and soil-transmitted helminthiasis (STH) are endemic diseases in Burundi. STH control is integrated into health facilities (HF) across the country, but schistosomiasis control is not. The present study aimed to assess the capacity of HF for integrating intestinal schistosomiasis case management into their routine activities. In addition, the current capacity for HF-based STH case management was evaluated.

**Methods:**

A random cluster survey was carried out in July 2014, in 65 HF located in *Schistosoma mansoni* and STH endemic areas. Data were collected by semi-quantitative questionnaires. Staff with different functions at the HF were interviewed (managers, care providers, heads of laboratory and pharmacy and data clerks). Data pertaining to knowledge of intestinal schistosomiasis and STH symptoms, human and material resources and availability and costs of diagnostic tests and treatment were collected.

**Findings:**

Less than half of the 65 care providers mentioned one or more major symptoms of intestinal schistosomiasis (abdominal pain 43.1%, bloody diarrhoea 13.9% and bloody stool 7.7%). Few staff members (15.7%) received higher education, and less than 10% were trained in-job on intestinal schistosomiasis case management. Clinical guidelines and laboratory protocols for intestinal schistosomiasis diagnosis and treatment were available in one third of the HF. Diagnosis was performed by direct smear only. Praziquantel was not available in any of the HF. The results for STH were similar, except that major symptoms were more known and cited (abdominal pain 69.2% and diarrhoea 60%). Clinical guidelines were available in 61.5% of HF, and albendazole or mebendazole was available in all HF.

**Conclusions:**

The current capacity of HF for intestinal schistosomiasis and STH detection and management is inadequate. Treatment was not available for schistosomiasis. These issues need to be addressed to create an enabling environment for successful integration of intestinal schistosomiasis and STH case management into HF routine activities in Burundi for better control of these diseases.

**Electronic supplementary material:**

The online version of this article (10.1186/s40249-018-0447-y) contains supplementary material, which is available to authorized users.

## Multilingual abstracts

Please see Additional file 1 for translation of the abstract into the six official working languages of the United Nations.

## Background

Among the neglected tropical diseases (NTD), schistosomiasis and soil-transmitted helminthiasis (STH) are the most common parasitic infections. Both have major implications for health and socioeconomic aspects and are important public health problems in Burundi [1].

From 1950 to the 1960s, population movement from the highlands to the Rusizi plain, deterioration of water and sanitation infrastructures and migration of refugees from neighbouring countries contributed to a serious increase of the disease burden due to *Schistosoma mansoni* [2–6]. A high prevalence of STH (*Ascaris lumbircoides*, *Trichuris trichuria*, and hookworm) in Usumbura in 1935 [7] and in the Central Highlands (around Kitega) in 1936 [8] had already been reported.

In Burundi, STH case management has been integrated into health facilities (HF) across the country for many decades. This is not the case for schistosomiasis, for which any form of control only began in the 1970s. In 1973, a schistosomiasis treatment programme for schools was implemented in the capital city of Bujumbura, and ambulant treatment for positive cases was initiated at the health centres (HC). In both cases, direct smear was used for diagnosis and niridazole for treatment [6, 9]. This resulted in a decrease of the school prevalence of *S. mansoni* infection from 16% in 1974 to 10% in 1982 [9]. With the availability of new tools for diagnosis (Kato-Katz test) and treatment (praziquantel [PZQ]) [10] of intestinal schistosomiasis, a new national control programme was launched in 1982 for intestinal schistosomiasis and STH control using Kato-Katz as the diagnostic tool and PZQ and mebendazole (MBZ) for intestinal schistosomiasis and STH treatments, respectively [9]. Ten years later, in the primary schools of Bujumbura, the prevalence had decreased from 49.5 to 29.4% for STH and from 23.3 to 6.4% for *S. mansoni* infection [9].

**Fig. 1:**
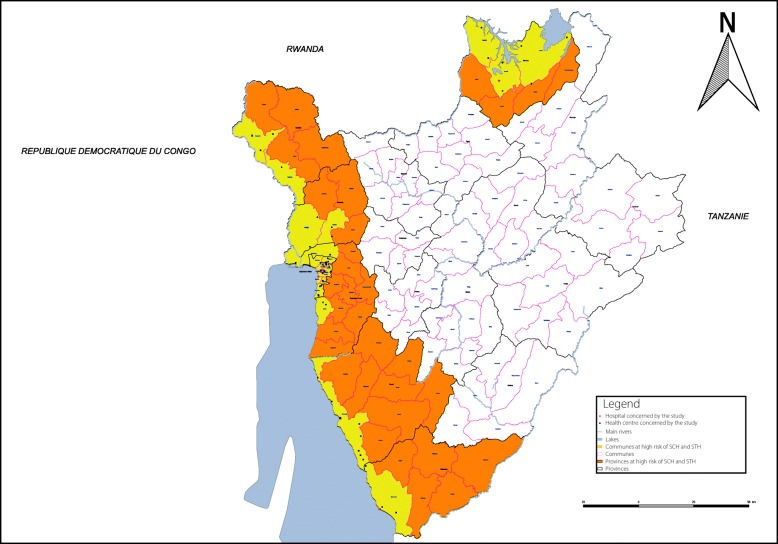
Burundi map indicating the location of the 65 health facilities included in the study, Burundi 2014. SCH: Schistosomiasis; STH: Soil-transmitted helminthiasis.

In 1989, *S. mansoni* control was integrated into the primary health care (PHC) services of all provinces of Burundi where the disease was endemic (Bujumbura Mairie, Bujumbura Rural, Bubanza, Cibitoke, Bururi, Makamba and Kirundo) [11] (Fig. 1). However, in 1993, the civil war began. Throughout the country, the resulting situation of instability affected all factors of life, including the health sector, and control activities declined considerably [11]. In 2005, *S. mansoni* and STH were again widespread, with prevalence in some provinces reaching 61 and 60%, respectively [12].

In 2007, a Neglected Tropical Disease Control Programme was implemented in Burundi. The main objectives were to define populations at risk for each NTD and to develop and implement a drug treatment strategy targeting at-risk populations. A nationwide school-based survey in 20 schools confirmed that intestinal schistosomiasis and STH were still endemic in the country, with 24 *communes* at high risk of these diseases [13]. No urogenital schistosomiasis was found, confirming previous reports [3, 4]. During the same year, a programme of mass drug administration (MDA) with PZQ and albendazole (ALB)/MBZ was initiated. Target groups were school-age children for PZQ and children between 1 and 14 years of age and pregnant women for ALB/MBZ [13]. From 2007 to 2011, the treatment coverage in school-age children at the national level increased from 87.9% in 2008 to 95.9% in 2011 [13].

Data from a countrywide reassessment of *S. mansoni* infection in Burundi published in 2017 showed a significant decrease in the prevalence from 12.7% in 2007 to 2.2% in 2014 [14] in *communes* targeted by MDA [14]. For STH, a significant decrease was also registered from 2007 to 2014 [15]. The pooled STH prevalence was 32% in 2007 and 18% in 2014 [15].

In Burundi, STH control is integrated into HF across the country, but schistosomiasis control is not, with only HC involved and only during MDA campaigns. Vertical programmes are difficult to maintain over long periods, especially when they are dependent on external funding [16, 17]. For sustainability reasons, the World Health Organization (WHO) 1993 recommendation on integrating schistosomiasis control into PHC services [18] was reconsidered by others [19] in 2010.

We investigated the capacity of HF to integrate intestinal schistosomiasis case management into their routine activities. In addition, the capacity for current HF-based STH case management was evaluated. To this end, the current knowledge of health staff on the symptoms and the available options for diagnosis and treatment of intestinal schistosomiasis and STH were assessed in endemic areas in Burundi at HF of different levels.

## Material and methods

### Burundi: Health system and general socioeconomic context

In Burundi, the health system consists of three levels. The central level consists of the directorate-general, departmental directorates, vertical programmes and national and specialized hospitals. The intermediate level is composed of 17 sanitary provinces (SP) corresponding to the administrative provinces. At the peripheral level, there are 45 sanitary districts (SD) with 753 HC and 63 hospitals. Each SD includes a district hospital (DH) and many HC. The SD covers an average of two or three *communes*. A *commune* is the most decentralized and operational unit of Burundi’s administrative system. Burundi had 129 *communes* in 2014, at the time of the survey. The size of a *commune* was 216 km^2^ (average), with an average population of 71 978 inhabitants per *commune*. The HC is the first patient contact point in the health system and is generally managed by a nurse. The heads of the SP, SD and DH are medical doctors.

Most of the Burundian population live in precarious socioeconomic conditions, with 8 out of 10 living below the poverty level (less than 1 USD/day) [20]. The per capita daily income is 0.64 USD and 0.41 USD in urban and rural areas, respectively. The gross domestic product (GDP) fell dramatically from 286 USD/capita in 1993 to 176 USD/capita in 2011 [21]. Eighty percent of the population live less than 5 km from HC; however, 17% of patients do not have access to care, and 81.5% of patients are obliged to borrow money or sell assets to cope with health/medical expenses [20]. Furthermore, only 55% of households have access to clean water in rural areas and 85% in urban areas [20], while only 12.6% use latrines or pit latrines [20]. Sixty-one percent of the HC and 27% of the primary schools have tap water. Ninety percent of the population attend primary school, but only 17 and 24% of girls and boys, respectively, complete the first level of secondary school, and only 9 and 17% complete the second level [21].

### Study area

This study was conducted in 24 *communes* endemic for intestinal schistosomiasis and STH in Burundi. These *communes* are located in the following seven provinces: Bujumbura Mairie, Bujumbura Rural, Bubanza, Bururi, Cibitoke, Kirundo and Makamba. These provinces are located in the west, south and north of Burundi. All are close to lakes and rivers separating Burundi from the Democratic Republic of Congo in the west (Lake Tanganyika and the Rusizi River), Tanzania in the south and east (Maragarazi River) and Rwanda in the north (Lake Cohoha and Lake Rweru) (Fig. 1).

### Study design

This study was designed to assess the knowledge of health staff on the symptoms and the available options for diagnosis and treatment of intestinal schistosomiasis and STH in endemic areas of Burundi. While the whole country is endemic for STH infections, only 24 *communes* are considered to be at risk of intestinal schistosomiasis infection (per 2007 mapping) [13]. Based on these data, 65 HF were randomly selected from a list of 220 HF located in those 24 *communes* endemic for intestinal schistosomiasis and STH. Selection was performed via proportional allocation to strata (*communes*), ensuring that at least one HF in each *commune* was randomly selected.

### Questionnaires and data collection

In each of the 65 HF, five staff were interviewed: the person in charge of the HF (manager), the person in charge of consultations and referral for patients (care provider), the person in charge of the laboratory (head of laboratory), the person in charge of the pharmacy (head of pharmacy), and the person in charge of case reporting (data clerk). The interviewers were medically trained people. A pre-survey was carried out in six HC (not included in the final randomization of the main survey) in the 13 *communes* of Bujumbura Mairie to test the questionnaires in terms of formulation and understanding of the questions.

The survey was conducted in July 2014, from the 5th to 12th and was organized into 3 axes and nine teams with, respectively, three supervisors and nine team leaders, supervised by one coordinator. The questions posed to the HF staff focused on (i) HF type (public, *confessional* and private) and level (national hospital, regional hospital, DH and HC) in the Burundian health pyramid; (ii) knowledge and education/training of staff (degree/diploma and position held, in-job training on intestinal schistosomiasis and STH and knowledge of symptoms related to intestinal schistosomiasis and STH); (iii) diagnosis (use and availability of clinical guidelines, laboratory protocols and diagnostic tests for intestinal schistosomiasis and STH); (iv) treatment (availability, purchasing and supply process and the list of essential medicines); (v) costs (consultation fees and prices for diagnosis and treatment); and (vi) case reporting activities (monitoring of patients on treatment and reporting of diagnosis and treatment to the National Health Information System [NHIS]).

#### Definition of some degrees/diplomas used in the questionnaires in the Burundian context

A0 = Secondary school + 4 years of higher education (higher education is defined as the superior level that follows secondary school; secondary school = high education); A1 = Secondary school + 3 years of higher education; A2 = Primary school + 8 years of secondary school; A3 = Primary school + 6 years of secondary school.

### Statistical analysis

**Fig. 2:**
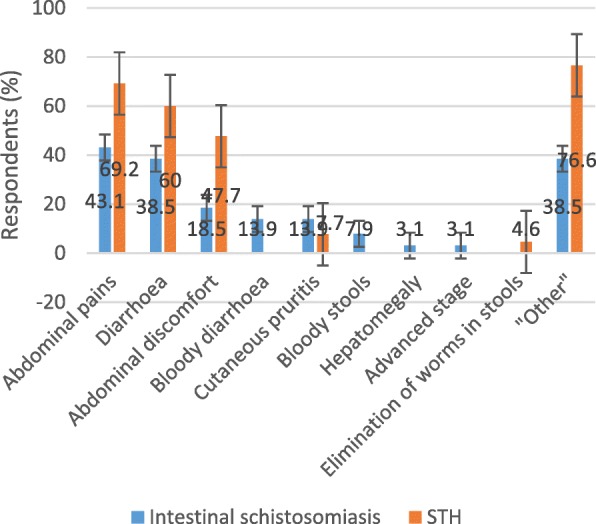
Symptoms related to intestinal schistosomiasis and soil-transmitted helminthiasis, as mentioned by care providers in 65 health facilities. For intestinal schistosomiasis, “Other” refers to other (non- or less-specific) symptoms: fever, headache, “ballonnement abdominal” (bloated feeling, flatulence and swollen abdomen), nausea and vomiting. “Advanced stage” is related to symptoms for advanced disease such as oedema, ascites and haematemesis. For soil-transmitted helminthiasis (STH), “Other” refers to other (non-specific) symptoms: chest pain, low back pain, fever and loss of weight

Databases were created in Excel 2013 (Microsoft, Redmond, United States of America) and exported to Stata version 12 (StataCorp. LP, College Station, United States of America) for statistical analysis. Data are shown in tables and graphs with calculated frequencies (with 95% confidence intervals for proportions in Fig. 2) and mean ± standard deviation (or median and interquartile range (IQR) when the distribution was abnormal) for qualitative and quantitative variables, respectively. Chi square and Fisher’s exact tests were used to analyse the association of the knowledge of care providers on different symptoms of schistosomiasis and STH with their level of education, using an α risk error of 5% (*P* < 0.05).

## Results

### General characteristics of HF

Among the 65 HF selected, 61 were HC (94%) and four were hospitals. Among the 61 HC, 22 (33.9%) were public, nine (13.9%) *confessional* and 30 (46.2%) private. Among the four hospitals, two (3%) were public district hospitals, one was a public National Hospital (1.5%) and one was private (1.5%).

### Human resources

#### Education and job type of staff

Table 1 gives an overview of the education and job type of the HF staff. Managers (43.1%) and care providers (46.2%) were mainly A2 nurses, while heads of pharmacy (36.9%) were mainly A3 nurses. For data clerks, the proportions of A2 and A3 nurses were equal (38.5%). Heads of laboratory were mainly A2 laboratory technicians (52.3%).

**Table 1:** Education and job type of interviewees in 65 of 220 health facilities in Burundi in 2014

Education and job type	Manager of HF (Percentage)	Care Provider (Percentage)	Head of laboratory (Percentage)	Head of pharmacy (Percentage)	Data clerks (Percentage)
MD Specialist	2 (3.0%)	0 (0.0%)	0 (0.0%)	0 (0.0%)	0 (0.0%)
MD General Practitioner	5 (7.7%)	9 (13.8%)	0 (0.0%)	0 (0.0%)	2 (3.1%)
Pharmacist	1 (1.5%)	0 (0.0%)	0 (0.0%)	1 (1.5%)	0 (0.0%)
Nurse A0	2 (3.1%)	1 (1.5%)	1 (1.5%)	0 (0.0%)	2 (3.1%)
Laboratory Technician A0	0 (0.0%)	0 (0.0%)	5 (7.7%)	0 (0.0%)	0 (0.0%)
Midwife A0	0 (0.0%)	1 (1.5%)	0 (0.0%)	0 (0.0%)	1 (1.5%)
Nurse A1	6 (9.2%)	4 (6.1%)	0 (0.0%)	2 (3.1%)	7 (10.8)
Laboratory Technician A1	0 (0.0%)	0 (0.0%)	1 (1.5%)	0 (0.0%)	0 (0.0%)
Nurse A2	28 (43.1%)	30 (46.2%)	9 (13.8%)	21 (32.3%)	25 (38.5%)
Laboratory Technician A2	0 (0.0%)	0 (0.0%)	34 (52.3%)	2 (3.1%)	1 (1.5%)
Nurse A3	21 (32.3%)	19 (29.2%)	5 (7.7%)	24 (36.9%)	25 (38.5%)
Laboratory Technician A3	0 (0.0%)	0 (0.0%)	5(7.7%)	0 (0.0%)	0 (0.0%)
Other	0 (0.0%)	1 (1.5%)	7(10.8%)	15 (23.1%)	2 (3.1%)
Total	65	65	65	65	65

*HF* Health facilities, *MD* Medical doctor; A0 = Secondary school + 4 years of higher education (higher education is defined as the superior level that follows secondary school; secondary school = high education); A1 = Secondary school + 3 years of higher education; A2 = Primary school + 8 years of secondary school; A3 = Primary school + 6 years of secondary school

Across all levels, the number of staff with a higher education was very low. Only 15.7% had a higher education (A1, A0, pharmacist and medical doctor), while 76.6% had secondary education in health sciences, mainly A2 and A3 nurses.

#### Training on intestinal schistosomiasis and STH

Only 8.1 and 9.2% of the interviewees received training on intestinal schistosomiasis and STH, respectively. Less than 50% of trained staff received the training material to be used for reference in the field—38.1 and 45.8% for intestinal schistosomiasis and STH, respectively. The duration of the training ranged from one to seven days, except for the laboratory technicians, who were trained for up to 21 days. In more than 98% of cases, both STH and schistosomiasis training was given simultaneously.

#### Knowledge of symptoms of intestinal schistosomiasis and STH by care providers

Figure 2 shows all reported symptoms for intestinal schistosomiasis and STH. The knowledge level of care providers for intestinal schistosomiasis was very low. Bloody diarrhoea and bloody stools were mentioned as the main symptoms of intestinal schistosomiasis, at 13.9 and 7.7%, respectively. Abdominal pain for intestinal schistosomiasis was mentioned by 43.1% of the care providers as another important symptom. Knowledge of diarrhoea as a main symptom of intestinal schistosomiasis was related to the level of education and was significantly less among A3 diploma holders (10.5%) compared to superior diploma holders (50%) (Fisher’s exact test = 0.004).

For STH, the knowledge of symptoms was better than that for intestinal schistosomiasis. Abdominal pain was cited by 69.2% and diarrhoea by 60% of the care providers.

### Material resources

#### Clinical guidelines and laboratory protocols

The results of our study showed limited availability of material resources for clinical and laboratory diagnosis: 40 (61.5%) and 22 (33.8%) of the 65 HF had clinical guidelines for STH and intestinal schistosomiasis, respectively. Of these HF, only eight (36.4%) had general guidelines, and only two (9.1%) had a diagnostic algorithm to manage STH and intestinal schistosomiasis. Laboratory protocols on intestinal schistosomiasis and STH diagnosis were only available in 19 (29.2%) and 24 (36.9%) of the HF, respectively. Among these HF, only 21 (87.5%) and 4 (22.2%) had written SOPs (standard operating procedures) on the direct smear test and the Kato-Katz test, respectively.

#### Laboratory tests used for the diagnosis of intestinal schistosomiasis and STH

In all 65 HF, only a direct smear test was available for laboratory diagnosis of intestinal schistosomiasis and STH, which was applied in 58 (90.6%) and 64 (98.5%) of the HF, respectively. The lack of some accessories was cited as one of the reasons of this situation.

### Drug supply and availability of treatment

Among the heads of pharmacy, 36 (55.4%) reported receiving the drugs bought by HF in time, and 34 (52.3%) receiving them in the right quantity. Only 45 (69.2%) of the HF were allowed by the Ministry of Public Health to supply drugs of any kind. While ALB/MBZ and PZQ are on the list of essential drugs [22], this was recognized by the pharmacy staff only for ALB/MBZ, not for PZQ. In contrast to ALB/MBZ, which was present in every HF, PZQ was not available at any level. Thirty (46.2%) of the pharmacy staff reported that PZQ should be available in the HF, and 14 (46.7%) reported having the financial means to procure it. All 65 (100%) heads of pharmacy recognized that drugs used during the mass treatment campaigns (which are done twice and once a year for STH and schistosomiasis, respectively) are given by donors (Schistosomiasis Control Initiative [SCI] ranks first for drugs used for mass treatment campaigns, whether for STH or schistosomiasis).

### Financial accessibility to consultation, laboratory diagnosis and treatment

Table 2 gives details on the median costs with the IQR. Consultation and diagnosis (direct smear) costs were 2 times more expensive in private HC than in public and *confessional* HC and 5.8 times more expensive in private hospitals than in public and *confessional* hospitals. The treatment for STH was 2.4 times more expensive in private HC than in public and *confessional* HC. During the MDA campaigns, medication for STH and schistosomiasis was donated. In that case, the treatment was provided free of charge in public and *confessional* HC. For hospitals, the treatment was 1.3 times more expensive in private hospitals than in public and *confessional* hospitals.

**Table 2:** Median costs^a^ of schistosomiasis and soil-transmitted helminthiasis health care in 65 of 220 health facilities, Burundi 2014

	Public and *confessional* hospital	Private hospital	Public and *confessional* HC	Private HC
	(IQR)	(IQR)	(IQR)	(IQR)
People ≥5 years of age				
Consultation ticket ≥5 years	0.32(0.32–1.03)	6.47	0.06(0.03–0.19)	0.32(0.06–0.32)
Laboratory test for the diagnosis (direct smear)	1.13(0.65–1.13)	1.94	0.26(0.19–0.32)	0.32(0.26–0.32)
Praziquantel tablets	–	–	–	–
Albendazole tablets	0.08(0.05–0.26)	0.10	0.08(0.03–0.19)	0.19(0.19–0.32)
Pregnant women and children < 5 years of age^b^				
Consultation ticket	0.00	6.47	0.00	0.32(0.06–0.32)
Laboratory test for the diagnosis (direct smear)	0.00	1.94	0.00	0.32(0.26–0.32)
Praziquantel tablets	–	–	–	–
Albendazole tablets	0.00	0.10	0.00	0.19(0.19–0.32)
Total costs for schistosomiasis	1.45	8.41	0.32	0.64
Total costs for STH	1.53	8.51	0.40	0.83

*IQR* Interquartile range, *HC* Health centre, *STH* Soil-transmitted helminthiasis, − Not applicable

^a^Costs are in USD; 1 USD = 1546.6800 BIF (Burundian Franc) on the 2nd of July, 2014

^b^Pregnant women and children less than 5 years of age do not pay for health care in public and confessional HF

### Case reporting activities

According to the data clerks, the reporting of cases to the NHIS was functional for 63 (96.9%) of the HF visited, both for intestinal schistosomiasis and STH. An identical paper template was used in all HC and transmitted monthly to the NHIS. The hospitals also used reporting forms, but STH was not specified. Reports were transmitted to NHIS only by the DH, not by the national and private hospitals.

Follow-up with the patients under STH treatment was reported to be done in 49 (75.4%) of the 65 HF. However, the follow-up visits were actually registered in only 23 of these (46.9%). For schistosomiasis, there was no treatment available and, thus, no follow-up.

## Discussion

We aimed to assess the capacity of HF to integrate intestinal schistosomiasis case management into routine activities. We also evaluated the capacity for current STH case management, which has been integrated into the HF for many decades. We found the following: (i) Few staff members had received higher education, and even less had received training on intestinal schistosomiasis case management. The level of knowledge of the main symptoms of intestinal schistosomiasis was low and was related to the level of education. (ii) Clinical guidelines and laboratory protocols were available in only one third of the HF. (iii) In HF with laboratory facilities, only direct smear was used to diagnose intestinal schistosomiasis. (iv) PZQ was not available in any of the HF. (v) Consultation and diagnosis (based on direct smear) were relatively expensive, considering the socioeconomic conditions of Burundians. The results for STH were similar, except that (i) the level of knowledge of main symptoms was considerably higher, (ii) clinical guidelines were available in a higher proportion of the HF (61.5%), and (iii) ALB/MBZ was available in all HF.

The number of HF staff with higher education was very low in our study. This problem is not specific to Burundi. It is a reality in low-income countries [23] that only few HF are managed by medical doctors. For example, in Kenya [24], clinical officers in DH were reported to perform medical tasks that are typically assigned to medical doctors, including medical surgery.

Even though in-job training cannot replace prior education [25], it can bring added value in strengthening the capacity of HF for the prevention and control of infectious diseases [19, 26–32]. In Mali [33] and Senegal [34], training on schistosomiasis case management was provided as part of an intervention project on curative health care, and knowledge of health care providers about the symptoms of *S. mansoni* infections was high (69–94%) after the training. This is not the case in Burundi, where the percentage of staff that received specific training on intestinal schistosomiasis diagnosis and treatment was very low.

Previous studies have shown that inadequate supply [35] and expired medicines and other materials [30, 35], plus shortages of essential drugs, are access barriers to health services, especially in rural areas [30]. The drug supply problems identified in this study have been observed in other countries as well, such as Ethiopia [25], Uganda and Tanzania [29]. Studies in Ghana [36] and Senegal [34] reported PZQ to be out of stock in 22.5 and 25% of HF, respectively, obliging patients to be referred to other HF or to seek treatment elsewhere. In Burundi, PZQ was unavailable at any HF, causing a major constraint for integration of schistosomiasis case management into HF [37]. Similarly, the lack of adequate diagnostic tests has also been observed in other countries [33, 36] and can seriously hamper intestinal schistosomiasis and STH detection and management in HF. The WHO recommends using Kato-Katz testing for the diagnosis of intestinal schistosomiasis [18] and STH, which is more sensitive than direct smear and still relatively cheap [18]. Recent research has reported the circulating cathodic antigen test in urine as a more sensitive test [14, 38, 39] for intestinal schistosomiasis, in addition to being easy to use. However, it is more expensive than the Kato-Katz test [40].

In Burundi, the average per capita daily income is below the poverty level (1USD): 0.64 USD per day and 0.41 USD per day in urban and rural areas, respectively [21]. Our results show that the costs of consultation and diagnosis for intestinal schistosomiasis/STH range from 0.32 to 8.41 USD, depending on the HF type and level. Hence, many Burundians cannot access care without sacrificing other needs. At present, only pregnant women and children under five years of age do not pay for health care in public or *confessional* HF. To remove the financial barrier to health care, this should be extended to other vulnerable groups as well.

## Conclusions

This study highlighted the lack of capacity in Burundian HF for integrating intestinal schistosomiasis case management into their routine activities. For STH, the capacity for HF-based case management was better, but it still needs improvement. Especially at the HC level, which is the first point of contact with the community, there is an urgent need for the government to address these capacity gaps in order to provide adequate diagnosis and treatment for intestinal schistosomiasis and STH for everybody at all times. At present, treatment of intestinal schistosomiasis is limited to MDA for school-age children [13], and PZQ is not available outside of the yearly campaigns or for other target groups, jeopardizing accessibility and equity of services [19]. In addition, diagnosis in HF of both intestinal schistosomiasis and STH is currently based on direct smears in the context of poor knowledge of symptoms among care providers and, thus, is sub-optimal. As a consequence, many cases are not diagnosed and/or not treated, causing the spread of these infections and potential severe complications in the long term. HF capacity strengthening for case management is an essential, albeit not the only, requirement for sustainable control of intestinal schistosomiasis and STH in Burundi and elsewhere.

## Additional file

Additional file 1: Multilingual abstracts in the six official working languages of the United Nations. (PDF 648 kb)

## Abbreviations

ALB: Albendazole
BIF: Burundian Franc
DH: District Hospital
GDP: Gross Domestic Product
HC: Health Centre
HF: Health Facility
IQR: Interquartile Range
MBZ: Mebendazole
MD: Medical Doctor
MDA: Mass Drug Administration
NHIS: National Health Information System
NTD: Neglected tropical diseases
*P*: *P*-value
PHC: Primary Health Care
PZQ: Praziquantel
SCH: Schistosomiasis
SCI: Schistosomiasis Control Initiative
SD: Sanitary District
SOP: Standard Operating Procedure
SP: Sanitary Province
USD: United States dollar
WHO: World Health Organization
