# Supplementary material for: Capacity gaps in health facilities for case management of intestinal schistosomiasis and soil-transmitted helminthiasis in Burundi
Source: Infect Dis Poverty. 2018 Jul 4;7:66. doi: 10.1186/s40249-018-0447-y (PMC6030799; doi:10.1186/s40249-018-0447-y)
Supplement: Supplementary file 1 — Multilingual abstracts in the six official working languages of the United Nations. (PDF 648 kb) [file 40249_2018_447_MOESM1_ESM.pdf]

Translation of the abstract into the six official working languages of the United Nations

الفجوات فى قدرات المنشآت الصحية لكيفية التعامل مع البلهارسيا المعوية وداء الديدان المنتقل عن طريق التربة فى بوروندي .

قدمه: بول بيزيمانا ، وكاتيا بولمان ، وجان بيير فان جيرترودين ، وفريدريك نسابيومفا ، وسيلين نجينزبوهورو ، وألفيس موهيمبوندو ، وجيسيبينا أورتو

الملخص

المعلومات الأساسية: تعتبر البلهارسيا وداء الديدان التي تنتقل عبر التربة (HST) من الأمراض المستوطنة فى بوروندي . ويتم ادماج مكافحة داء الديدان المنتقل عبر التربة فى المنشآت الصحية فى جميع أنحاء البلاد ولكن لا يشمل ذلك مكافحة داء البلهارسيا . تهدفُ الدراسة الحالية إلى تقييم قدرة المنشآت الصحية على دمج كيفية التعامل مع حالات البلهارسيا المعوية كجزء من أنشطتها الروتينية . وإضافةً إلى ذلك، تم تقييم قدرة المنشآت الصحية التى تتعامل مع داء الديدان المنتقل عبر التربة .

الأساليب: أُجريت بدراسة استقصائية عشوائية متعددة في شهر تموز 2014 فى 65 منشأة صحية موجودة فى المناطق التى يستوطن فيها فيروس البلهارسيا المعوية وداء الديدان المنتقل عبر التربة . وجُمعت المعلومات عن طريق استبيانات تحتوى على أسئلة شبه كمية. أُجريت مقابلات مع الموظفين باختلاف مهامهم فى المنشآت الصحية (المدراء ومقدمى الرعاية ورؤساء المختبر والصيدلية وكتبة إدخال البيانات) . كذلك جُمعت المعلومات المتعلقة بما يعرف عن البلهارسيا المعوية وداء الديدان المنتقل عبر التربة والموارد البشرية والمادية وعن توافر وتكاليف الفحوصات التشخيصية والعلاج .

النتائج : أقل من نصف عدد مقدمى الرعاية وعددهم 65 ذكروا واحدًا أو أكثر من أعراض الإصابة بمرض البلهارسيا المعوية (ألم فى البطن 43.1% ، اسهال مصحوب بالدم 13.9% وبراز مصحوب بالدم 7.7%) عدد قليل من الموظفين (15.7%) كانوا من الحاصلين على التعليم العالى وأقل من 10% كانوا من الحاصلين على التدريب الوظيفى حول كيفية التعامل مع حالات البلهارسيا المعوية . وتوفرت المبادئ التوجيهية الطبية والبروتوكولات المتبعة فى المختبر بخصوص تشخيص وعلاج مرض البلهارسيا فى ثلث عدد المنشآت الصحية . وكان التشخيص يتم عن طريق أخذ عينات مباشرة فقط . ولم يتوفر دواء برازيكوانتيل فى أي من المنشآت الصحية. كانت النتائج بالنسبة لداء الديدان المنتقل عبر التربة مشابهة ، الا أن الاعراض الرئيسية للمرض كانت معروفة وتم ذكرها بصورة أكبر (ألم فى البطن 69.2% و الاسهال 60%). توفرت المبادئ التوجيهية الطبية فى 61.5% من المنشآت الطبية وكان دواء ألبيندازول وميبيندازول متوفر فى كل المنشآت الصحية .

االاستنتاجات : ب الات صال الم ن قولة الأمراض عن وال ك شف الـ معوي ة الـ ب لهار س يات ل داء دي دي ل لإد ش الـ حال ية وال قدرة ..كاف ية غ ير وإدارت ها الـ ج ن سيلا يتوفر علاج لمرض البلهارسيا. يجب معالجة هذه القضايا حتى يتم تكوين بيئة قادرة على ادماج صحيح لأساليب التعامل مع حالات البلهارسيا المعوية وداء الديدان المنتقل عبر التربة وتكون من ضمن النشاطات الروتينية للمنشآت الصحية فى بوروندي حتى تتم مكافحة هذه الأمراض بطريقة أفضل.

Translated from English version into Arabic by Bashaier Allam, proofread by Free bird, through

TRANSLATORS
WITHOUT BORDERS

布隆迪卫生机构在肠道血吸虫病和土源性蠕虫病病例管理方面的能力差距

Paul Bizimana, Katja Polman, Jean-Pierre Van Geertruyden, Frédéric Nsabiyumva, Céline Ngenzebuhoro, Elvis Muhimpundu, Giuseppina Ortu

摘要

引言：血吸虫病和土源性蠕虫病（STH）是布隆迪的地方病。STH 防治工作被纳入全国各地卫生机构日常工作中，但血吸虫病防治未被纳入。本研究旨在评估卫生机构将肠道血吸虫病病例管理纳入为日常工作的能力，并对基于卫生机构的 STH 病例管理能力进行评估。
方法：2014 年 7 月对曼氏血吸虫和 STH 流行区的 65 个卫生机构进行随机聚类调查。通过半定量问卷收集数据。采访了卫生机构不同职能部门的工作人员（管理人员、护理人员、实验室和药房主管、数据文员），收集肠道血吸虫病和 STH 症状的相关知识知晓情况，人力物力资源，诊断测试和治疗的可及性和费用等相关数据。
结果：在 65 名护理人员中，不到一半的人员知晓肠道血吸虫病的一种或多种主要症状（腹痛 43.1%，血性腹泻 13.9%，血便 7.7%）。极少数工作人员（15.7%）接受过高等教育，只有不到 10%的人员接受过肠道血吸虫病病例管理的在职培训。1/3 的卫生机构有肠道血吸虫病诊断和治疗的临床指南和实验室规程。所有的卫生机构均是采用直接涂片法诊断血吸虫病。除了主要症状的知晓率和引用率更高（腹痛 69.2%、腹泻 60%）外，STH 的调查结果与肠道血吸虫病相似。61.5%的卫生机构有临床指南，所有的卫生机构都有阿苯达唑或甲苯咪唑，但没有吡喹酮。
结论：目前，卫生机构对肠道血吸虫病和 STH 检测和管理的能力不足，无法治疗血吸虫病。通过将肠道血吸虫病和 STH 病例管理成功纳入到布隆迪卫生机构的日常工作中，更好地为防控上述疾病创造有利环境，才能解决这些问题。

Translated from English version into Chinese by Translated by Pei Wang, edited by Pin Yang

CHINESE CENTER FOR DISEASE CONTROL AND PREVENTION
NATIONAL INSTITUTE OF PARASITIC DISEASES

Lacunes en matière de capacité dans les établissements de santé au Burundi pour la gestion des cas de schistosomose intestinale et d'helminthoses transmises par le sol

Paul Bizimana, Katja Polman, Jean-Pierre Van Geertruyden, Frédéric Nsabiyumva, Céline Ngenzebuhoro, Elvis Muhimpundu, Giuseppina Ortu

Résumé

Contexte: Les schistosomoses et les helminthoses transmises par le sol (HTS) sont des maladies endémiques au Burundi. Le contrôle des HTS est prescrit dans les établissements de santé (ES) à travers le pays, contrairement à celui des schistosomoses. La présente étude vise à évaluer la capacité des ES à intégrer la gestion des cas de schistosomose intestinale dans leurs activités de routine. D'autre part, la capacité actuelle de gestion des cas de HTS présents dans les ES a également été évaluée.
Méthodes: Une enquête aléatoire par grappes a été réalisée en juillet 2014 dans 65 ES situés dans des zones où *Schistosoma mansoni* et les HTS sont endémiques. Les données ont été recueillies à l'aide de questionnaires semi-quantitatifs. Les membres du personnel de l'ES occupant différentes fonctions ont été interrogés (directeurs, personnel soignant, directeurs de laboratoire, pharmaciens en chef et secrétaires). Les données recueillies ont porté sur la connaissance des symptômes de la schistosomose intestinale et des HTS, les ressources matérielles et humaines, la disponibilité ainsi que le coût des tests diagnostiques et des traitements.

Résultats: Moins de la moitié des 65 soignants connaissent un ou plusieurs symptômes de la schistosomose intestinale (douleurs abdominales 43,1 %, diarrhée sanguinolente 13,9 % et selles sanguinolentes 7,7 %). Un faible pourcentage des membres du personnel (15,7 %) ont fait des études supérieures et moins de 10 % ont été formés à la gestion des cas de schistosomose intestinale. Des directives cliniques et les protocoles de laboratoire pour le diagnostic et le traitement de la schistosomose intestinale étaient disponibles dans un tiers des ES. Le diagnostic n'était réalisé que par frottis direct. Aucun ES n'avait de praziquantel. Les résultats étaient similaires pour les HTS, à l'exception des principaux symptômes qui étaient mieux identifiés et cités (douleurs abdominales 69,2 % et diarrhée 60 %). Des directives cliniques étaient disponibles dans 61,5 % des ES, l'albendazole ou le mébendazole était disponible dans tous les ES.
Conclusions: La capacité actuelle des ES pour la détection et la prise en charge de la schistosomose intestinale et des HTS est inadaptée. Il n'y avait pas de traitement disponible pour les schistosomoses. Pour un meilleur contrôle de ces maladies au Burundi, ces problèmes doivent être résolus afin de créer un environnement propice à une bonne intégration de la gestion des cas de schistosomose intestinale et des HTS dans les activités de routine des ES.

Translated from English version into French by Céline Cloarec, proofread by Suzanne Assenat, through

Нехватка ресурсов в медицинских учреждениях для лечения случаев кишечного шистосомоза и геогельминтоза в Бурунди

Пол Бизимана, Катя Польман, Жан-Пьер Ван Гертруйден, Фредерик Нсабиюмва, Селин Нгензебухоро, Элвис Мухимпунду, Джузеппина Орту

Аннотация

Сведения для справки: Шистосомоз и геогельминтоз - эндемические заболевания в Бурунди. В отличие от геогельминтоза, лечение шистосомоза не проводится в медицинских учреждениях (МУ) страны. Цель настоящего исследования - оценить, возможно ли включить в работу МУ лечение случаев кишечного шистосомоза. Кроме того, в рамках исследования были оценены текущие возможности лечения случаев геогельминтоза в МУ.
Методы: В июле 2014 года было проведено кластерное рандомизированное исследование в 65 МУ, находящихся в эндемических очагах *шистосомоза Мансона* и геогельминтоза. Сбор данных осуществлялся с помощью полуколичественных вопросников. Опрос проводился среди сотрудников МУ различных должностей (управляющие, медицинские работники, руководители лабораторий и аптек, канцелярские работники). Были собраны данные по осведомленности о симптомах кишечного шистосомоза и геогельминтоза, о людских и материальных ресурсах, а также о наличии и стоимости диагностических тестов и лечения.

Результаты: Менее половины из 65 медицинских работников смогли назвать хотя бы один из основных симптомов кишечного шистосомоза (боль в животе - 43,1%, кровавый понос - 13,9% и кровянистый стул - 7,7%). Очень немногие из работников (15,7%) имели высшее образование, и менее 10% прошли курс по лечению случаев кишечного шистосомоза. Клинические рекомендации и лабораторные протоколы для диагностики и лечения кишечного шистосомоза имелись лишь в трети МУ. Диагностика проводилась только по результатам нативного мазка. Празиквантел не имелся в наличии ни в одном МУ. Результаты опроса по геогельминтозу были сходными, однако основные симптомы этого заболевания оказались более известными и назывались чаще (боль в животе - 69,2%, диарея - 60%). Клинические рекомендации имелись в 61,5% МУ. Во всех МУ имелся в наличии альбендазол либо мебендазол.
Заключение: На сегодняшний день возможности диагностики и лечения кишечного шистосомоза и геогельминтоза в МУ являются неудовлетворительными. Возможности для лечение шистосомоза отсутствуют. Решение этих проблем создаст благоприятные условия для успешной интеграции практики лечения кишечного шистосомоза и геогельминтоза в деятельность бурундийских МУ для борьбы с этими заболеваниями.

Translated from English version into Russian by Polina Nikitina, proofread by Tatiana Kary, through

TRANSLATORS WITHOUT BORDERS

Déficit de recursos en los centros sanitarios de Burundi para la coordinación asistencial de la esquistosomiasis intestinal y la helmintiasis transmitida a través del suelo

Paul Bizimana, Katja Polman, Jean-Pierre Van Geertruyden, Frédéric Nsabiyumva, Céline Ngenzebuhoro, Elvis Muhimpundu, Giuseppina Ortu

Resumen
Introducción: La esquistosomiasis y la helmintiasis transmitida a través del suelo (STH, por sus siglas en inglés) son endemias en Burundi. La lucha contra la STH está integrada en los servicios sanitarios del país, pero no se combate la esquistosomiasis. Este estudio tiene como objetivo analizar la capacidad de los servicios sanitarios para integrar en sus actividades habituales la coordinación asistencial de la esquistosomiasis intestinal y la STH.
Metodología: Se realizó un estudio aleatorizado por conglomerados en julio de 2014 en 65 centros sanitarios situados en zonas endémicas de *Schistosoma mansoni* y STH. Los datos se recogieron mediante cuestionarios semicuantitativos y se interrogó a diferentes miembros del personal de los centros sanitarios (directores, profesionales sanitarios, jefes de laboratorio, jefes de farmacia y administrativos). Se recopilaron datos sobre sus conocimientos acerca de los síntomas de la esquistosomiasis intestinal y la STH; los recursos humanos y materiales; y la disponibilidad y coste de las pruebas diagnósticas y del tratamiento.
Resultados: Menos de la mitad de los 65 profesionales sanitarios mencionó uno o más síntomas significativos de esquistosomiasis intestinal (dolor abdominal 43.1 %, diarrea hemorrágica 13.9 % y rectorragia 7.7 %). Pocos

(15.7 %) habían cursado estudios superiores y menos del 10 % habían recibido formación en su puesto de trabajo sobre la coordinación asistencial de la esquistosomiasis intestinal. Un tercio de los centros sanitarios disponía de directrices clínicas y protocolos de laboratorio para el diagnóstico y tratamiento de la esquistosomiasis intestinal. El diagnóstico se realizaba exclusivamente mediante frotis directo y ningún centro disponía de praziquantel. Los resultados para la STH fueron parecidos, aunque el personal conocía mejor los síntomas significativos y los mencionó en mayor medida (dolor abdominal 69.2 % y diarrea 60 %). El 61.5 % de los centros sanitarios disponía de directrices clínicas y todos los centros tenían acceso a albendazol o mebendazol.

Conclusiones: Actualmente, los centros sanitarios no están capacitados para detectar y tratar la esquistosomiasis intestinal y la STH. No había ningún tratamiento disponible para la esquistosomiasis. Para controlar mejor estas enfermedades, es necesario abordar estos problemas con el objetivo de crear un entorno que permita integrar con éxito la coordinación asistencial de la esquistosomiasis intestinal y la STH en las actividades habituales de los centros sanitarios de Burundi.

Translated from English version into Spanish by Lidia Norese, proofread by Maria CG, through

TRANSLATORS
WITHOUT BORDERS
